# Effects of insecticides on mortality, growth and bioaccumulation in black soldier fly (*Hermetia illucens*) larvae

**DOI:** 10.1371/journal.pone.0249362

**Published:** 2021-04-21

**Authors:** Nathan Meijer, Theo de Rijk, Joop J. A. van Loon, Lisa Zoet, H. J. van der Fels-Klerx

**Affiliations:** 1 Wageningen Food Safety Research, Wageningen, The Netherlands; 2 Plant Sciences Group, Laboratory of Entomology, Wageningen University, Wageningen, The Netherlands; 3 Bestico B.V., Berkel en Rodenrijs, The Netherlands; Institut Sophia Agrobiotech, FRANCE

## Abstract

Residues of persistent insecticides may be present in the substrates on which insects are reared for food and feed, which may affect insect growth or survival. In addition, insecticidal substances may bio-accumulate in reared insects. The objective of this study was to assess potential effects of selected insecticides on the growth and survival of black soldier fly larvae (BSFL, *Hermetia illucens*) and on their safety when used as animal feed. Six insecticides (chlorpyrifos, propoxur, cypermethrin, imidacloprid, spinosad, tebufenozide) with different modes of action were tested in two sequential experiments. Cypermethrin was also tested with the synergist piperonyl butoxide (PBO). Standard BSFL substrate was spiked to the respective maximum residue level (MRL) of each insecticide allowed by the European Union to occur in feed; and BSFL were reared on these substrates. Depending on the observed effects in the first experiment, spiked concentrations tested in the second experiment were increased or reduced. At the concentrations applied (1 and 10 times MRL), three of the six tested substances (chlorpyrifos, propoxur, tebufenozide) did not affect the survival or biomass growth of BSFL, compared to the control (non-spiked) treatments. At MRL, imidacloprid stimulated the growth of BSFL compared to the controls. Spinosad and cypermethrin at the MRL level negatively affected growth and survival. The effects of cypermethrin appeared to be augmented by addition of PBO. A mean bio-accumulation factor of ≤0.01 was found in both experiments for all substances–except for cypermethrin, which was comparatively high, but still below 1 (0.79 at 0.1 mg/kg). The lack of accumulation of insecticides in the larvae suggests that there is no risk of larval products being uncompliant with feed MRLs. However, we conclude that insecticides present in substrates may affect growth and survival of BSFL. More research on a larger variety of substances and insect species is recommended.

## Introduction

Insects as food and feed are increasingly seen as a commercially viable mini-livestock alternative to conventional livestock [1]. Larvae of the black soldier fly (BSFL, *Hermetia illucens* (L.); Diptera: Stratiomyidae) had primarily been seen as a pest species [2], but have recently received increased attention given their ability to consume and convert a wide variety of organic waste, including vegetable and animal tissue, and manure [3,4]. However, several safety aspects, including the effects of contaminants that could be present in the feed substrates, have received less attention. One concern is that bio-accumulation of contaminants or chemical residues could result in exceedance of legal limits, or even present a safety risk, when insects–exposed via the substrate on which they are reared–are consumed as food or feed [5]. Synthetic insecticides are an important category amongst potential contaminants that was highlighted for additional research [5,6]. Moreover, since persistent synthetic insecticides act against all insects, residues of these insecticides present in the substrate may cause (sub-)lethal effects in insects produced for food and feed, even at concentrations in the feed substrate that comply with existing legal limits.

The legal framework for pesticides in the European Union (EU) consists of Regulation (EC) No 1107/2009 concerning the placing of plant protection products [i.e. pesticides] on the market, and Regulation (EC) No 396/2005 on maximum residue levels (MRLs) of pesticides in or on food and feed of plant and animal origin. Maximum levels for certain substances, primarily those prohibited from use in the EU, are set at the lower limit of analytical quantification (LOQ). A pesticide is only approved after risk assessment on its effects on honeybees (*Apis mellifera* L.; Hymenoptera: Apidae*)*, *Bombus* spp. (Hymenoptera: Apidae) and solitary bees has been performed; and it has been concluded that its use would result in negligible exposure of honeybees, and that there will be no unacceptable acute or chronic effects on colony survival and development (Regulation (EC) No 1107/2009, Annex II, section 3.8.3; EFSA, 2013 [7]). Since insects intended for food or feed are farmed animals (Regulation (EU) No 2017/893), the substrates on which they are reared also must comply with the legal frameworks for pesticides and animal feed (Article 3 of Regulation (EC) No 1069/2009). However, we are not aware of any regulations that consider the potential effects of insecticide residues on insects reared for food and feed in the approval and MRL-setting procedures.

Research on the effects of pesticides on insects has largely focused on two categories of species. Firstly, much research has been done on the eco-toxic effects of insecticides on beneficial species, in particular honeybees (*Apis mellifera* L.; Hymenoptera: Apidae) due to its well-studied biology, and the mentioned legal requirements for (re-)approval of pesticides [8]. A second strand of research has focused on pest species as target of the tested substances [9]. However, dose-response relationships are in principle species-specific [10], preventing the extrapolation of (sub-)lethal effects of insecticides on other species to BSFL.

The literature on the impact of pesticides on growth and survival of BSFL specifically appears to be relatively scarce. Recent controlled feeding studies on the effects of pesticide residues in the substrate of BSFL have been performed by Tomberlin et al. (2002) (cyromazine, pyriproxifen, λ-cyhalothrin, permethrin) [11], Lalander et al. (2016) (azoxystrobin, propiconazole) [12], and Purschke et al. (2017) [13] (chlorpyrifos, chlorpyrifos-methyl, pirimiphos-methyl) [13]. In none of the studies on BSFL mentioned, accumulation of pesticides was observed, and the tested substances did not appear to negatively affect BSFL at concentrations in the feed at or below the MRL.

The primary aim of this study was to perform exploratory research on the effects of a variety of insecticides on the larval stage of *Hermetia illucens*. We hypothesized that residues of tested substances, when present in the feed substrate at concentrations equal to the legal limit in the EU, would affect the growth or survival of BSFL negatively; and that the substances would possibly bio-accumulate in the larvae, resulting in food and feed safety issues. The focus of the experiments was specifically on the larval stage (day 7–14) that is most interesting for commercial rearing of BSFL for feeding purposes.

## Methodology

### Choice of pesticides

Pesticides targeted at insects (insecticides) can be classified into different groups according to their chemical class and mode of action (MoA). The Insecticide Resistance Action Committee (IRAC) developed a classification scheme in which the insecticide target site and the physiological functions affected, i.e. nerve and muscle, growth, respiration, or mid-gut, were used as classification criteria [14,15].

The insecticides to be investigated in this study were chosen using a step-wise approach. A longlist of 54 insecticides, acaricides, and nematicides was created from a total of 527 compounds (including isomers) listed in the database of the Dutch National Monitoring Programme on contaminants in feed and food. The 54 insecticides included in the longlist were detected (i.e. analytical result > LOD) in feedstuffs and edible insects in The Netherlands, as based on data from the Dutch National Control Programme Animal Feed in 2015–2016, and data supplied by three major insect producing companies in The Netherlands. Of these, 16 substances were isomers of other substances and were therefore disregarded. Of the remaining 38 substances, one insecticide was selected from each chemical class/MoA as classified by IRAC [14]. In case of multiple insecticides per class, the substance with the highest number of entries in the Rapid Alert System for Food and Feed (RASFF) database for the years 2015–2017 was selected. In total, six insecticides and one synergist were selected. These selected compounds, their class and mode of action, are summarized in Table 1.

**Table 1:** Selected insecticides including class, mode of action, and maximum residue level (MRL) in the EU for feed, or maize specifically.

Substance name	Class	Mode of Action	MRL^1^ (mg/kg)
Chlorpyrifos	Organophosphates	Acetylcholinesterase (AChE) inhibitors	0.05
Propoxur	Carbamates	Acetylcholinesterase (AChE) inhibitors	0.05*
Cypermethrin	Pyrethroids	Sodium channel modulators	0.3
Imidacloprid	Neonicotinoids	Nicotinic acetylcholine receptor (NAchR) competitive modulators	0.1
Spinosad	Spinosyns	Nicotinic acetylcholine receptor (NAchR) allosteric modulators–site I	2.0
Tebufenozide	Insect growth regulators (IGRs)	Ecdysone receptor agonists	0.05*
Piperonyl butoxide	Synergist	Synergist	Not a plant protection product; no MRL

1: For feed (Directive 2002/32/EC), if available, or maize (Reg. (EC) No 396/2005).

*: Indicates lower limit of analytical determination.

The insecticides methoxychlor (MoA: sodium channel modulator) and various isomers of DDT were detected (>LOQ) in feed materials in the Netherlands National Control Programme Animal Feed 2015–2016. However, these have not been included in the final list because they have been banned in the EU since 2002 (Regulation (EC) No 2076/2002) and exposure is therefore expected to be incidental only. More RASFF notifications have been filed for the neonicotinoid acetamiprid (189) than for imidacloprid (76). However, due to imidacloprid’s higher toxicity and increased attention it has received recently due to its sub-lethal effects on honeybees (see e.g. EFSA (2018) [16]), it was decided to select imidacloprid instead of acetamiprid. The impact of the organophosphate chlorpyrifos on BSFL was found to have no effect on survival and growth, and not to bio-accumulate (0.4 mg/kg) by Purschke et al. (2017) [13]; its inclusion in this study was aimed to verify their results on the BSF strain we tested. At the time that the experiments reported here were conducted (September 2018), chlorpyrifos was still permitted to be used in the EU. In 2019, the European Food Safety Authority (EFSA) concluded that the genotoxic potential of chlorpyrifos could not be excluded, and it was therefore recommended that its approval would not be renewed [17]. With the substance’s authorisation expiring after 31 January 2020, this non-renewal effectively prohibited the use of this pesticide after this date (Regulation (EU) 2020/18).

Piperonyl butoxide (PBO) is not an insecticide in the strict sense of the word: it is primarily used as a synergist. Some studies suggest that PBO may have intrinsic insecticidal properties by acting as a juvenile hormone mimic (preventing adult development) [18]. However, it is generally used at sub-lethal levels to enhance the effects of mainly pyrethrin and synthetic pyrethroids [19]. In this study, PBO was therefore combined with cypermethrin in a single treatment, in addition to a treatment containing only PBO. No MRL has been set for PBO in the EU because it is not registered as a plant protection product (Regulation (EC) No 2016/2288).

### Feed preparation

The feed preparation and experimental set-up were largely based on Camenzuli et al. (2018) [20]. A standard substrate containing primarily wheat, potato, and yeast used for commercial rearing of BSFL was obtained from Bestico B.V. (Berkel en Rodenrijs, The Netherlands), where the experiments were conducted. Two experiments were performed with a number of treatments: in experiment 1 (Exp. 1), individual batches of feed were spiked to the European MRL for that insecticide in feed specifically (‘1*MRL’) as defined in Directive 2002/32/EC), or for maize (as defined in Regulation (EC) No 396/2005), see Table 1. Depending on the effects of the insecticides at these levels in terms of BSFL growth and mortality as compared to control, higher or lower concentrations were used in experiment 2 (Exp. 2, ‘+/-*MRL’; see below). Due to the short shelf-life of the substrate, spiked concentrations could not be verified prior to the experiment, nor checked for the presence of insecticides prior to spiking.

The analysed substances, their purity, solvent, and suppliers, are presented in Table 2. PBO was spiked in two treatments: one treatment in which it was spiked together with the pyrethroid cypermethrin at a ratio common in commercial formulations, of 20:1 [21], and one treatment containing only PBO–at the same concentration as in the other treatment. The spinosad treatment was a mixture of spinosyns A and D at a ratio of 74.2:22.3. In Exp. 2, the spiked concentration for cypermethrin was 1/3 * MRL (this also affected the PBO level since that was 20 * the level of cypermethrin). The spiked concentration of spinosad in Exp. 2 was 0.1 * MRL. Lower levels for these two insecticides in Exp. 2 relative to Exp. 1 were chosen due to the higher mortality observed in the spinosad treatment. For the other compounds (chlorpyrifos, propoxur, imidacloprid, and tebufenozide), the spiked concentration in Exp. 2 was 10 times the spiked concentration of Exp. 1, i.e. 10 * MRL.

**Table 2:** Insecticides tested, their intended spiked concentration in BSFL-feed, solvent, purity and suppliers.

Substance	Intended spiked concentration (mg/kg)		Solvent	Purity (%)	Supplier
	Exp. 1	Exp. 2			
Chlorpyrifos	0.05	0.5	MeOH	99.3	Sigma-Aldrich^1^
Propoxur	0.05	0.5	MeOH	99.9	HPC^2^
Imidacloprid	0.1	1.0	MeOH	98.7	HPC^2^
Spinosad	2.0	0.2	MeOH	96.6	HPC^2^
Tebufenozide	0.05	0.5	ACN	99.9	Sigma-Aldrich^1^
Cypermethrin	0.3	0.1	ACN	99.7	HPC^2^
Piperonyl butoxide (PBO)	6.0	2.0	ACN	92.5	Dr. Ehrenstorfer^3^
Cypermethrin + PBO	0.3 +6.0	0.1 +2.0	ACN	99.7 +92.5	HPC^2^ +Dr. Ehrenstorfer^3^

1: Sigma-Aldrich Chemie N.V., Postbus 27, 3330 AA Zwijndrecht, The Netherlands.

2: HPC Standards GmbH, Am Wieseneck 7, 04451 Cunnersdorf, Germany.

3: LGC Standards GmbH, Mercatorstrasse 51, 46485 Wesel, Germany.

In addition to the spiked feed treatments, three control treatments were used in Exp. 1: one blank, and two solvent controls (either acetonitril (ACN) or methanol (MeOH)). In Exp. 2, only the two solvent controls containing ACN and MeOH controls were used and the blank control was omitted because differences between the three controls in Exp. 1 were not significant.

Per treatment, 400 g (± 1 g) of feed was weighed in a 1 L beaker. This feed was spiked with the selected insecticide (dissolved in the respective solvent) to the desired concentration. The feed was then homogenized with a Bosch ErgoMixx hand-mixer (Robert Bosch Hausgeräte GmbH, Munich, Germany). From each beaker, 50 g (± 0.25 g) of spiked feed was transferred to each of the three containers in which the larvae would be placed. In addition, 1 g (± 0.1 g) of spiked feed was transferred into test tubes to verify the homogeneity of the insecticides in the spiked feed; and 50 g was placed in a separate container to verify the concentration. See section 2.4 for a description of the quality control (QC) parameters of these analyses.

### Animal procedures

Treatments were performed in triplicate. Per replicate, 100 seven-day-old larvae (post-hatching) were reared on 50 g of feed. The containers used in this trial were cylindrical (diam. 100 mm, height 40 mm) and a circular area (diam. 40 mm) in the centre of the lid consisted of fine mesh to allow for ventilation (SPL Life Sciences Co., Ltd., Gyeonggi-do, South Korea). Containers were distributed over trays; these trays were stacked and placed in a climate chamber (set at 28°C and 60% RH). The larvae were reared for another seven days until day 14 post-hatching, which was in line with the commercial practices of BSFL rearing at Bestico (Berkel en Rodenrijs, The Netherlands), where the experiments were conducted.

After seven days, the larvae were separated from the residual material (RM), which consisted of larval excreta and residual feed. The larvae were counted by manually removing them from the container, using metal tweezers. Dead larvae that appeared desiccated or immobile, were separated from live larvae. Larvae for which it was doubted if they were dead or displayed thanatosis were provisionally placed with the dead larvae until subsequent steps had finished for that replicate (approximately 2 min.). Larvae that had resumed moving were placed with the respective live larvae; otherwise they would be presumed dead. Larvae and residual materials were weighed. Larvae collected from the substrate were cleaned by depositing them in a standard plastic kitchen sieve, cleaned by rinsing them with running water to remove adhering residual material, and then dried gently using a paper towel. Between treatments, equipment (sieves and forceps) was rinsed and dried. Finally, the larvae were killed by freezing and kept frozen (at -18° C) until subsequent chemical analyses. Residual material was collected, weighed, and then stored in a clean plastic container at -18° C until analysis.

### Chemical analyses

Concentrations of tested insecticides in the substrate, larvae, and residual material were analysed using liquid chromatography-mass spectrometry (LC/MS-MS).

#### Extraction

For extraction of the active compounds from the larvae, the following procedure was followed. Frozen sample material, 1.0 g (± 0.05 g), was weighed into a tube. This was diluted by adding 5 ml of milliQ and 5 ml of acetonitrile + 1% acetic acid. The sample was homogenised by using an ultra-turrax machine until finely ground; 0.5 g of sodium acetate (ACS reag. Ph Eur. Emsure Merck) and 2 mg MgSO_4_ (GPR Rectapur VWR chemicals) was added, followed by vortexing for 30 s and centrifugation for 5 min at 3600 rpm (VWR Microstar 17). A volume of 2.5 ml was evaporated to < 0.5 ml and acetonitrile (Biosolve HPLC Supra Gradient 01203502) was added to bring the sample volume to 0.5 ml. This sample was mixed with 75 mg MgSO_4_, 12.5 mg C18 (Bakerbond Octadecyl (C18) 40 μm, JT Baker), 125 mg PSA (Bondesil-PSA 40 μm Agilent Technologies) + 25 μl 2 μg/ml PCB 198 (Ultra Scientific RPC-075S (diluted from 100 μg/ml > 2 μg/ml in hexane)), and vortexed for 30 s, and finally centrifuged for 5 min at 13.000 rpm (Thermo Scientific SL 40R Centrifuge). This was transferred to an LC vial and diluted where necessary. For extraction of the residual material, the same procedure was followed except that the homogenisation step using an ultra-turrax machine was replaced with 30 min of end-over-end mixing.

#### Analyses

A Waters ultra-high-performance liquid chromatography (UPLC) system (Waters, Etten-Leur, The Netherlands) and an Applied Biosystems Qtrap 6500 MS (Applied Biosystems Bleiswijk, The Netherlands) equipped with an electrospray (ESI) source were used. Separation was performed on a Acquity UPLC HSS T3, 1.8 μm, 2.1 x 100 mm column (Waters, Etten-Leur, The Netherlands) using a flow rate of 0.4 mL/min. The column temperature was maintained at 40°C. Eluent A was water (purified using a Milli QR system with a minimal specific resistance of 10 MΩ.cm^-1^, or water of a similar quality) containing 5 mM ammonium formate (> 99%, Sigma-Aldrich 17843) and 0.1% (v/v) formic acid 98–100% (EMSURE® ACS, Reag. Ph Eur (VWR 1.00264.1000)). Eluent B was water/methanol 5/95 (methanol: Biosolve 13683502 Absolute HPLC Supra Gradient) (v/v) containing 5 mM ammonium formate and 0.1% (v/v) formic acid. Total runtime was 12 min. The UPLC gradient started with 100% A for 1 min, was linearly increased to 100% B over 5 min, and kept at this percentage for 3 min. Finally, the gradient was switched to 100% A again over 0.5 min and equilibrated for 2.5 min before the next injection took place. The injection volume was 10 μL.

#### MS/MS conditions

ESI-MS/MS was performed using multiple reaction monitoring (MRM) in positive mode. Acquisition was done with 10 ms dwell time. The settling time and MR pause time was set to 5 ms. The number of data points across the peaks was at least eight. The settings of the ESI-source were as follows: source temperature 500°C, curtain gas 35 psi, source gas 1 50 psi, source gas 2 50 psi, ion spray voltage + 4000 V and collision gas (nitrogen) medium. The analyte-dependent parameters declustering potential (DP), collision energy (CE) and cell exit potential (CXP) are listed in Table in S1 Table.

#### Quality control

Quality control (QC) was performed by spiking blank samples with active substances in the range of 1–100 μg/kg. Results for these analyses can be found in Table in S2 Table (substrate), S3 Table (residual material) and S4 Table (substrate). For the residual material and larvae, the QC was performed for both experiments; for the substrate this was performed for Exp. 1.

### Calculations

Based on concentrations in the three matrices (substrate, larvae, and residual material), bio-accumulation and mass balance calculations were performed. The bioaccumulation factor (BAF) was defined as the concentration of the analysed insecticide in the larvae, divided by the concentration of that compound in the feed [22]. Bio-accumulation calculations for Exp. 1 were based on the analysed concentration in the substrate, whereas for Exp. 2, bio-accumulation calculations were estimates based on the spiked concentration in the substrate. In case the concentration in the larvae was below the limit of quantification (< LOQ), the BAF was not calculated.

For Exp. 1, mass balance calculations were performed in order to compare the insecticide levels pre- and post-experiments. The analysed weight (mg) of quantified substances in (i) the larvae and (ii) residual matter, post-experiments, was determined and expressed as a percentage of the measured weight (mg) of analysed substances in the feed, pre-experiments. As with bio-accumulation, the mass of substances in the larvae and residual material expressed as a percentage of the mass in the substrate was not calculated if the concentration in the respective matrix could not be quantified (concentration below LOQ). The sum of the insecticide amount (in weight units) in the larvae and residual material post-experiment being equal to the amount in the feed substrate pre-experiment implies that the total amount of spiked substance was recovered, and that no metabolic conversion has taken place. It was assumed that the concentration as determined in the sample of larvae or residual material was homogenously distributed and representative for the concentration in the entire replicate from which the sample was taken. For Exp. 2, mass balance calculations were based on the intended spiked concentration in the substrate.

### Statistical analyses

For the statistical analyses, the software SPSS Statistics for Microsoft Windows (version 25.0.0.2, IBM Corp., Armonk, NY, United States) was used. Because the treatments were performed in triplicate, tests on conformity to a distribution type were not warranted and non-parametric statistical tests were therefore used to determine statistical significance of findings.

To test whether the solvents (ACN and MeOH) had a significant effect on growth or survival compared to the blank control in Exp. 1; the distributions of the three control treatments were compared using a Kruskal-Wallis test (α = 0.05). If this was not the case, the controls in Exp. 1 (n = 9) and Exp. 2 (n = 6) were pooled for further statistical comparison with treatments containing active substances in each of the respective experiments.

For Exp. 1, the effects of all active substances on survival and growth of the larvae were tested for significant differences among treatments by using a Kruskal-Wallis test (α = 0.05). In Exp. 2, compared to Exp. 1, some active substances were used at higher concentrations (10x), and some were used at lower concentrations (1/3 and 1/10)–depending on the results of Exp. 1. The treatments in Exp. 2 containing active substances that were present in concentrations 10x that tested in Exp. 1 (chlorpyrifos, propoxur, imidacloprid, tebufenozide), were tested together with the pooled controls for significant differences by using a Kruskal-Wallis test (α = 0.05). This was also done for the treatments containing active substances present at 1/3 the concentration of Exp. 1 (cypermethrin, PBO, cypermethrin + PBO). For the single treatment in Exp. 2 that contained an active substance present at 1/10 the concentration of Exp. 1 (spinosad), significance of differences with the pooled controls was tested by using a Mann-Whitney U test (α = 0.05).

For both Exp. 1 and Exp. 2, if differences between treatments and controls in the Kruskal-Wallis test were significant (P < 0.05), then each treatment was compared separately to the grouped controls by using a Mann-Whitney U test. Because this post-hoc test involved multiple comparisons, a lower α value (α = 0.01) was used.

## Results

### Quality control

In the substrate of Exp. 1, the recovery of substances was within the acceptable range of 70–120% [23]; with the exception of propoxur (138%). These samples complied to the repeatability criteria (RSD ≤ 20%) though, and it was therefore decided to correct the concentrations for recovery. In the residual material, the average recovery of all compounds complied to the acceptability criteria of 70–120%, with the exception of spinosad (47% in Exp. 2) for which concentrations were accordingly corrected. The exact reason for this could not be identified, but this may have been due to the higher spinosad concentration in the samples, when compared to the concentration in the QC samples. In BSF larvae samples, the average recovery of all compounds complied to the acceptability criteria of 70–120% with the exception of PBO (64% in Exp. 1). This was likewise assumed to be due to differences between the QC and spiked concentrations. All compounds complied to the repeatability criteria (RSD ≤ 20%), with the exception of tebufenozide with an RSD of 33%. This did not affect the results since no quantifiable amount of tebufenozide was detected in the triplicate samples.

### Larval survival

Data of larval survival are shown in Table in S5 Table, for both experiments. In two replicates in Exp. 1 (tebufenozide (n = 104), blank control (n = 105)) survival was > 100%, which was assumed to be due to a counting error. These outliers were therefore corrected by assuming survival was 100%. The mean larval survival of all replicates of the three control treatments in Exp. 1 (ACN, MeOH, blank) was 99.4 ± 0.7% and not statistically different between the three control treatments (P = 0.056), we therefore pooled the control groups for subsequent statistical tests. Because the solvents did not have a significant effect on survival in Exp. 1, the controls were also pooled in Exp. 2.

**Fig 1:**
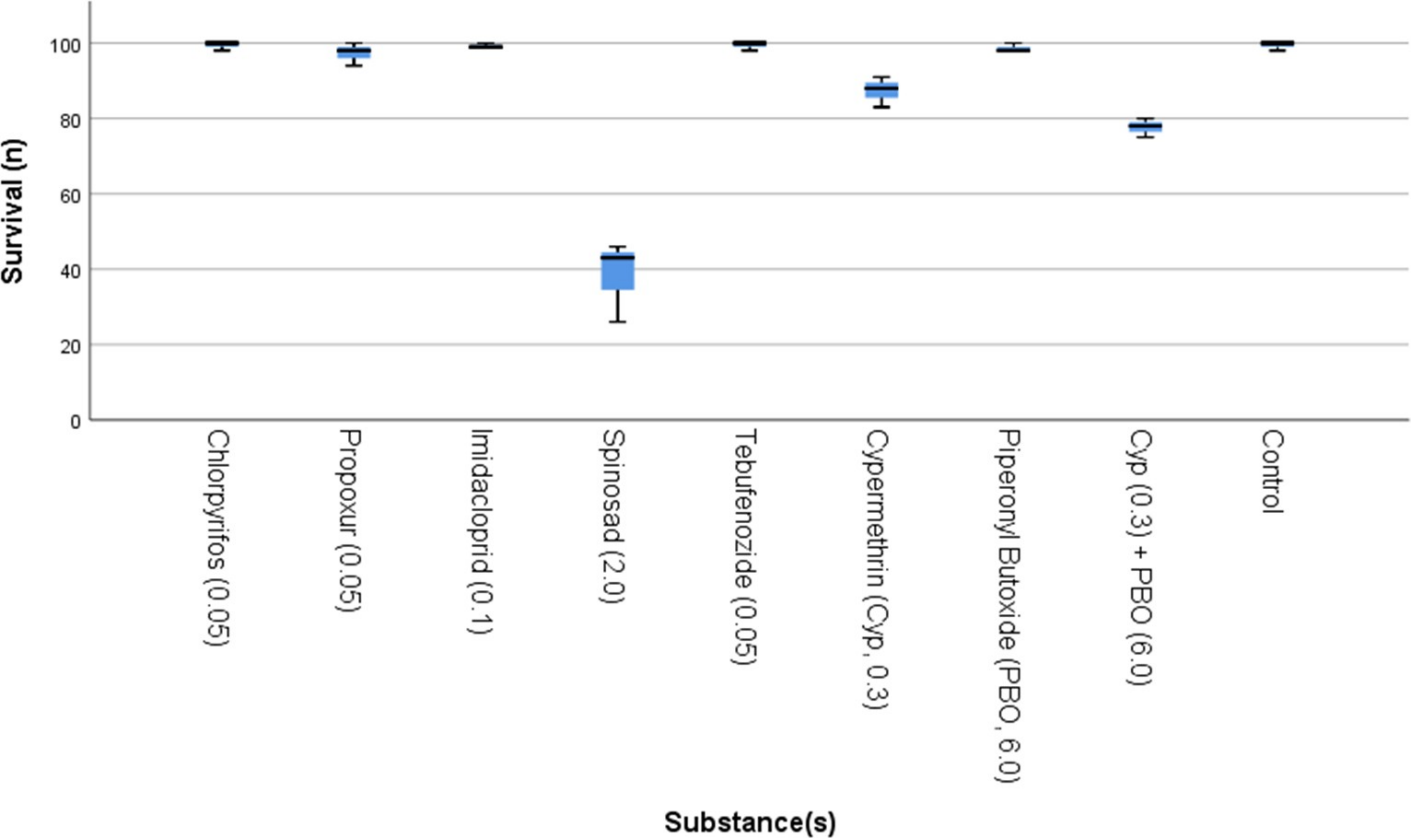
Box-plot of survival of black soldier fly larvae (*Hermetia illucens*) reared on substrates contaminated with different insecticides in Exp. 1. Results of the three control treatments have been grouped together. The concentration spiked in the substrate is indicated behind the name of the substance (mg/kg).

In Exp. 1, BSFL survival was statistically different between treatments (P = 0.004). The results for survival in Exp. 1 are shown in Fig 1. For most of the investigated insecticides (chlorpyrifos (P = 1.000), propoxur (P = 0.282), imidacloprid (P = 0.727), tebufenozide (P = 1.000), PBO without cypermethrin (P = 0.282)), differences in survival between these treatments and the control were not significant. However, spinosad (P = 0.009), cypermethrin (P = 0.009), and cypermethrin mixed with PBO (P = 0.009) reduced the survival of the larvae significantly as compared to the control in Exp. 1. The negative effect of cypermethrin on survival appeared to be enhanced by the addition of PBO, as compared to the treatment with cypermethrin only, but the number of replicates was too small to determine the significance of this difference using the Mann-Whitney U-test (n1 = n2 = 3).

In Exp. 2, differences between the controls and treatments containing active substances present in the substrate at 10x the concentration of Exp. 1 (chlorpyrifos, propoxur, imidacloprid, tebufenozide) were not significant (P = 0.155). This was also not significant for the substances present at 1/3 the concentration (cypermethrin, PBO, cypermethrin + PBO) (P = 0.356), nor for spinosad present at 1/10 the concentration (P = 1.000).

### Larval growth

Data on the increase in biomass of the larvae are shown in Table in S5 Table, for Exp. 1 and 2. The mean larval growth of all replicates of all control treatments in Exp. 1 was 10.86 ± 0.30 g and not statistically different between the three control treatments (P = 0.301), we therefore pooled the control groups for subsequent statistical tests. Because the solvents did not have a significant effect on survival in Exp. 1, the controls were also pooled in Exp. 2.

**Fig 2:**
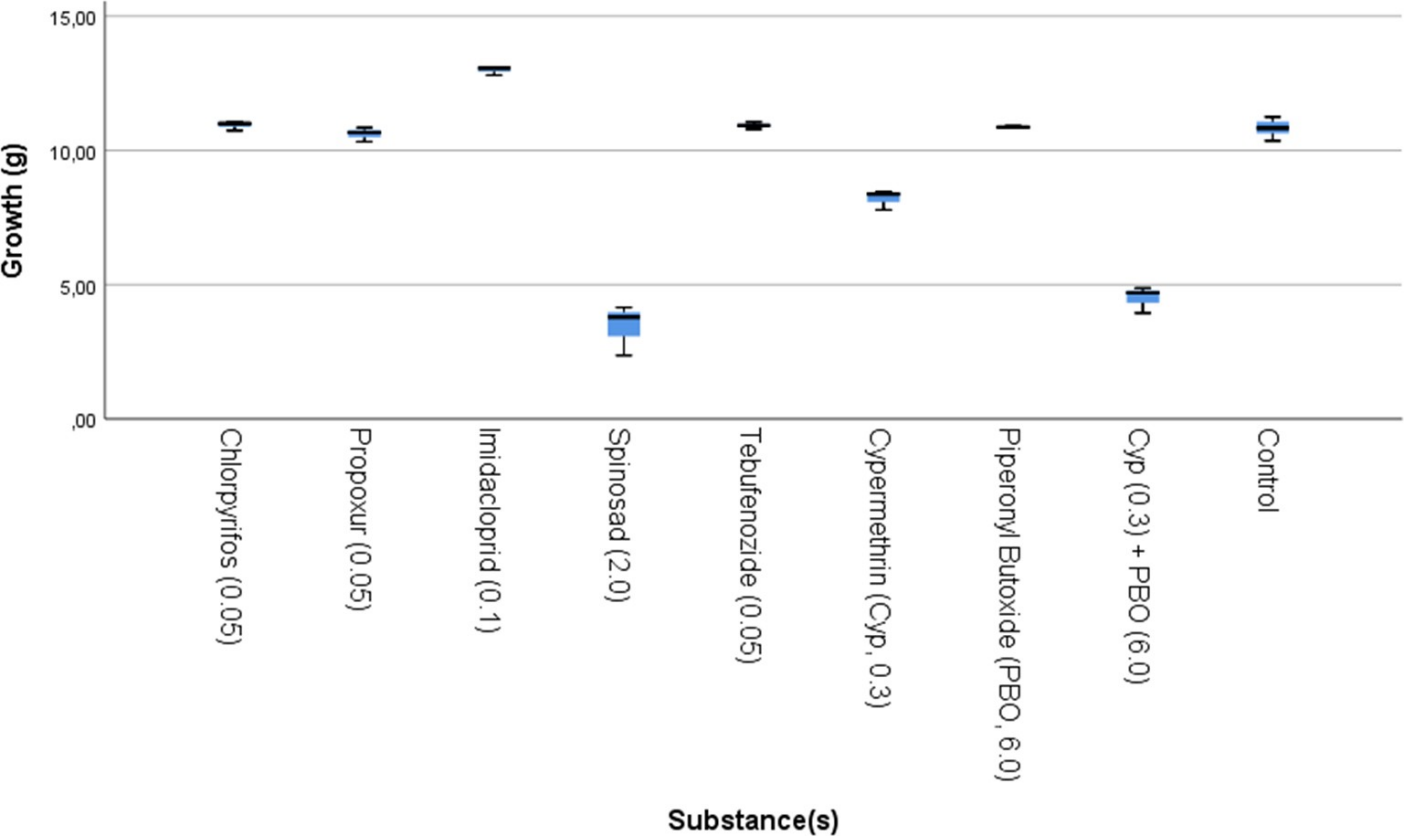
Box-plot of increase in biomass (g) of black soldier fly larvae (*Hermetia illucens*), reared on substrates contaminated with different insecticides in Exp. 1. Results of the three control treatments have been grouped together. The concentration spiked in the substrate is indicated behind the name of the substance (mg/kg).

As with survival, differences in larval growth were significant in Exp. 1 (P = 0.002). Results for the increase in biomass in Exp. 1 are shown in Fig 2; chlorpyrifos (P = 1.000), propoxur (P = 0.373), tebufenozide (P = 0.864), and PBO without cypermethrin (P = 0.864) did not affect larval biomass increase when compared to the control treatments (P > 0.01). However, imidacloprid significantly enhanced larval biomass growth (P = 0.009). The same insecticides that reduced larval survival (spinosad (P = 0.009), cypermethrin (0.009), and cypermethrin with PBO (P = 0.009)), also significantly reduced biomass increase compared to the control (P ≤ 0.01). As seen in the results for survival, PBO tended to enhance the negative effects of cypermethrin on growth but a test on significance could not be done due to low sample size (n1 = n2 = 3).

As seen for the survival in Exp. 2, differences in growth between treatments was not significant for any of the tested active substances. This was the case for those substances present in the substrate at 10x the concentration of Exp. 1 (chlorpyrifos, propoxur, imidacloprid, tebufenozide; P = 203); 1/3 the concentration (cypermethrin, PBO, cypermethrin + PBO; P = 0.069); and 1/10 the concentration (spinosad; P = 0.381).

### Concentrations and bioaccumulation

In the residual material of Exp. 1, cypermethrin (mean 0.035 mg/kg) and PBO (mean 0.013 mg/kg) in control treatments could be quantified in treatments in which it was not spiked. In Exp. 2, the mean concentrations of these two substances were 0.005 and 0.021 mg/kg, respectively. Other pesticide residues were not detected above their respective LOQ in treatments in which they had not been spiked.

The analysed concentrations (mg/kg) of tested compounds in the substrate (Exp. 1), and larvae and residual material (Exp. 1 and Exp. 2) are shown in Table in S6 Table (Exp. 1) and Table in S7 Table (Exp. 2).

**Fig 3:**
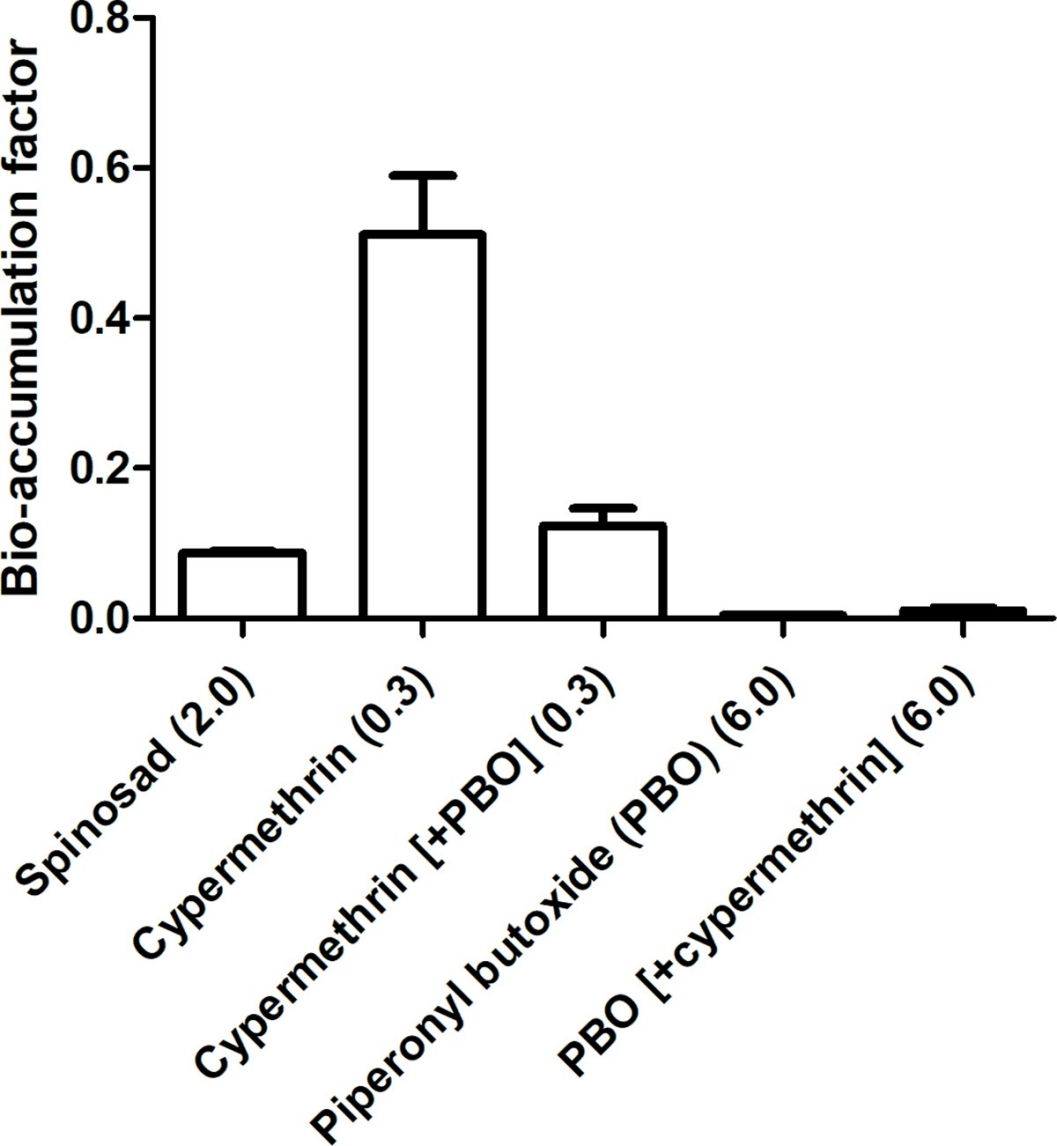
Bio-accumulation factor (mean + SD) of black soldier fly larvae (*Hermetia illucens*) reared on substrates contaminated with different insecticides in Exp. 1. The concentration spiked in the substrate is indicated behind the name of the substance (mg/kg).

Based on the analysed concentrations in Exp. 1, the BAF could be calculated for spinosad, cypermethrin, and PBO (shown in Fig 3). Concentrations of the remaining four substances (chlorpyrifos, propoxur, imidacloprid, tebufenozide) could not be quantified in the larvae, and the BAF was therefore not calculated. In case of cypermethrin, BAF was 0.51 ± 0.08; for other substances the mean BAF was < 0.20. Noteworthy is that the BAF of cypermethrin when mixed with PBO (0.12 ± 0.02) was four times lower than without this synergist (0.51 ± 0.08). Nonetheless, for all tested substances, mean BAF was < 1—signifying that none of these substances accumulated in the larvae.

In Exp. 2, the BAF was expressed as the analysed concentration in the larvae relative to the spiked concentration in the substrate. For this experiment, the BAF of cypermethrin was 0.79 ± 0.25 (without PBO) and 0.52 ± 0.19 (with PBO). While the concentration in the larvae could be quantified for the remaining substances (with the exception of propoxur), the mean BAF in Exp. 2 was ≤ 0.03 for each of these substances.

**Fig 4:**
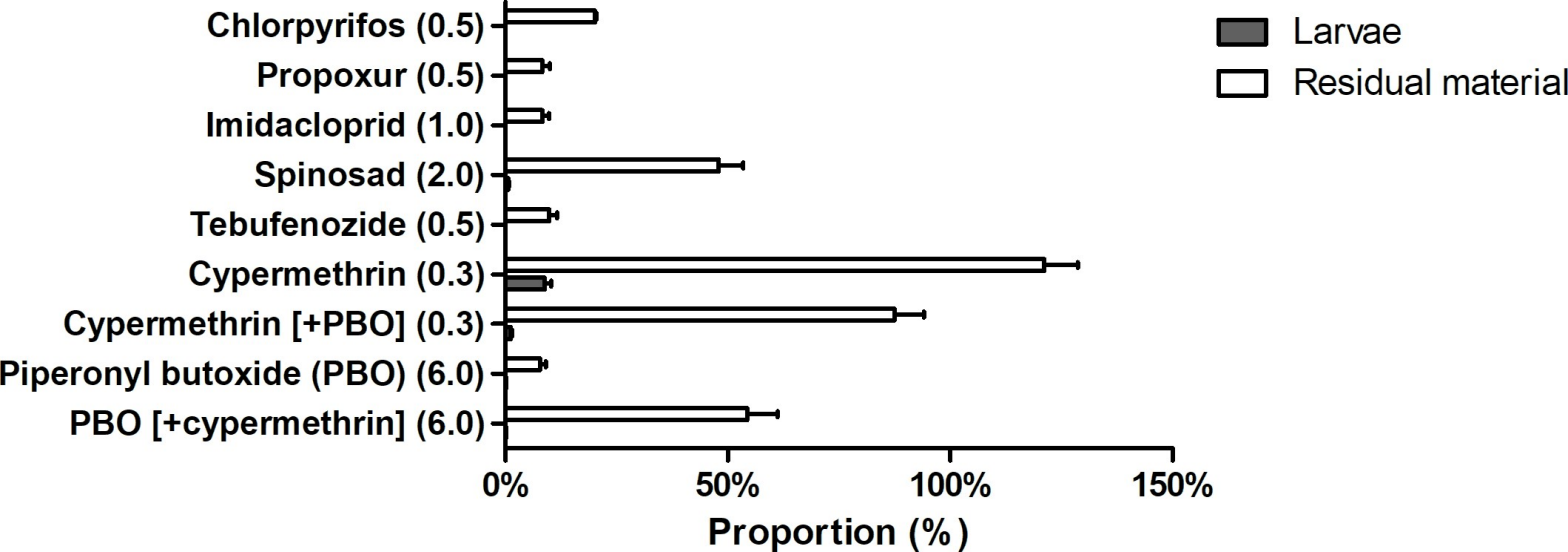
Mass balance (mean + SD) of different insecticides in Exp. 1 (n = 3 per treatment). Mass in black soldier fly larvae (*Hermetia illucens*) and residual material post-experiment expressed as a percentage of the analysed mass in the pre-trial substrate.

Mass balance calculations were performed for Exp. 1 (shown in Fig 4). As mentioned for the bio-accumulation, concentrations in the larvae could not be quantified for chlorpyrifos, propoxur, imidacloprid, and tebufenozide. In the residual material, the proportion of these substances post-trial was low (≤ 10%, and 20% for chlorpyrifos). Concentrations of spinosad and PBO could be quantified in the larvae, but the contributions to the total post-trial mass of each substance were very low (< 1.0%). The post-trial proportion of PBO in the residual material, relative to the respective total pre-trial mass in the substrate, was *ca*. seven times higher in the treatment also containing cypermethrin (54.3 ± 6.8%) than in the treatment without cypermethrin (7.8 ± 1.3%). The opposite was observed for cypermethrin, both in the larvae and the residual material. For both matrices, the proportion in the treatment containing only cypermethrin (larvae: 8.9 ± 1.4%; residual: 121.2 ± 7.5%) were on average higher than for the treatment containing both cypermethrin and PBO (larvae: 1.2 ± 0.3%, *ca*. 7 times lower; residual: 87.4 ± 6.7% (n = 2), 1.4 times lower). We ascribed the high concentration of cypermethrin in the residual material in Exp. 1 to inhomogeneous distribution of this substance in the matrix.

In Exp. 2, the mean proportion of substances in the larvae and residual material, as expressed as a percentage of the spiked mass, was low (< 10%) for chlorpyrifos, imidacloprid, tebufenozide, propoxur and PBO with and without cypermethrin. For all these substances, the percentage found back in the larvae was very low (< 1%). The proportion of spinosad post-trial was 13.4 ± 2.9%, with a higher percentage in the residual material (12.9 ± 2.8%) compared to the larvae (0.5 ± 0.2%). The proportion of cypermethrin recovered in the larvae, expressed as a percentage of the spiked amount, was 16.3 ± 5.2% (without PBO) and 10.9 ± 4.2% (with PBO). The concentration of cypermethrin in the residual material in the treatment without PBO was not determined due to a dilution error in sample preparation–but in the treatment with PBO it was also elevated at 22.2 ± 3.3%, compared to the other substances.

## Discussion and recommendations

Three of the tested active substances (chlorpyrifos, propoxur, tebufenozide) did not significantly affect growth nor survival, when present in the substrate at concentrations up to 10 times the MRL (0.5 mg/kg, for all three substances). Concentrations of these active substances in the larvae were either below the LOQ or very low, in comparison to the concentration in the substrate; indicating that bio-accumulation did not occur. For chlorpyrifos, these results confirm the findings of Purschke et al. (2017): in that study it was also concluded that there was no significant effect of chlorpyrifos spiked in the substrate at approximately the same concentration (0.4 mg/kg) on the survival and growth of BSFL, and that this substance did not bio-accumulate [13].

Mass balance calculations showed that total post-trial recovery of these insecticides in the larval biomass and residual material was below 100% in both experiments, compared to what was present in the substrate pre-trials. The mass balance being below 100% suggests that metabolic conversion of the spiked active substances may have occurred to some degree. Since growth and survival of BSFL were unaffected in these treatments, this conversion may have resulted in detoxification of the spiked parent compounds, but additional research on metabolic pathways in BSFL is needed to verify this.

Although we observed no effects on growth and survival of chlorpyrifos, propoxur, tebufenozide on BSFL in the specific developmental phase investigated in this study (7 to 14 days old), additional and/or different effects on adults or younger larvae cannot be excluded. In particular, sub-lethal effects of larval exposure on, *inter alia*, adult mating behaviour, fecundity, and longevity of adults [8] should be investigated to ensure that these substances do not affect the continuity of a colony, as this could also have major financial consequences for BSFL farmers.

Two of the tested insecticides (spinosad and cypermethrin) negatively affected both the growth and survival of the larvae when present at concentrations equal to the MRL (2.0 and 0.3 mg/kg, respectively). However, when lower concentrations of these substances were used in Exp. 2 (spinosad: 0.2 mg/kg; cypermethrin: 0.1 mg/kg), growth and survival of the BSFL were not affected to a significant degree.

The effects of cypermethrin appear to have been augmented by addition of the synergist piperonyl butoxide (PBO). In general, PBO’s mode of action–when combined with a pyrethroid such as cypermethrin–is that it inhibits microsomal oxidase enzymes from detoxifying the active substance [24]. Therefore, a higher concentration of cypermethrin would be expected to remain unmetabolized in the insects when they are also exposed to PBO, than if that synergist is not added. However, the opposite was observed in this study. The mean proportion of cypermethrin in the larvae recovered post-trial (16.2 ± 5.2%) was highest in the treatment without PBO in Exp. 2 in which survival and growth were not significantly affected–but lowest in the treatment containing both cypermethrin and PBO in Exp. 1 (1.2 ± 0.3%), in which survival and growth were lowest of the four tested treatments containing cypermethrin in the two experiments. These results taken together suggest that the interaction between cypermethrin and PBO that caused the reduced growth and survival, is linked to an as yet unknown metabolization of these substances to potentially more toxic metabolites. Additional research on the effects of cypermethrin in combination with PBO on BSFL is therefore recommended: to statistically validate the synergistic effects of PBO by using a higher number of replicates, and to determine the metabolic products and pathways that underlie these effects.

It is plausible that insecticides with the same MoAs as spinosad (nicotinic acetylcholine receptor (nachr) allosteric modulators—site I) and cypermethrin (sodium channel modulators) [15] may also negatively affect growth and survival of BSFL. An insecticide with the same MoA as spinosad is spinoteram; examples of other pyrethroids with the same MoA as cypermethrin include deltamethrin, cyfluthrin, and various isomers of these compounds [15]. Based on the findings presented here, we would advise prioritization of these substances in future studies on the effects of insecticide residues on BSFL.

Imidacloprid had a significantly positive effect on the increase in biomass in Exp. 1, compared to the control treatments. The positive effects of imidacloprid may be ascribed to insecticide-induced hormesis, a dose-dependent phenomenon in which low doses of a substance may incite stimulatory effects while higher doses are toxic [25]. Effects may include, amongst others, increase in birth rate, growth rate, and percentage of reproductive adults [26,27]. No prior studies published in the scientific literature on the effects of imidacloprid specifically on BSFL could be found, but there is some literature available on beneficial effects of sublethal doses of imidacloprid on other species. For instance, direct and intra-generational stimulatory effects on reproduction have been observed for sub-lethal doses of imidacloprid on green peach aphid (*Myzus persicae* Sulzer; Hemiptera: Aphididae) [28–30] and melon aphid (*Aphis gossypii* Glover; Hemiptera: Aphididae) [31]. Furthermore, hormetic effects were observed for reproduction and immature development duration of *Aphis glycines* Matsumura (Hemiptera: Aphididae) [32]; as well as an increase in reproductive fitness of male neotropical stink bugs (*Euschistus heros* F.; Heteroptera: Pentatomidae) [33]. It should be noted that the sublethal dose of imidacloprid that was observed to stimulate growth of the BSFL in this experiment, may also result in inhibitory effects on, for instance, pupation or reproduction, or possibly intra-generational effects [26]. Therefore, additional research on the dose-response relationship of imidacloprid and BSFL is highly recommended.

The findings from this study have implications for BSFL farmers and policy-makers. Insecticide MRLs depend on the feed material: for instance, the MRL of cypermethrin in maize that was used in this study is 0.3 mg/kg–while it is 2.0 mg/kg in wheat. Consequently, higher concentrations are permitted in wheat, which could have even more devastating effects on survival and growth than what was found in our experiments. Cypermethrin and spinosad are insecticides approved for use in the EU. Feed intended for insects with concentrations of these compounds at or slightly below the MRL may be legally put on the market–but at these concentrations they may pose a health risk to BSFL, and thus present a commercial risk for the insect farmer. It is recommended that BSFL farmers target cypermethrin and spinosad in analyses of their incoming feed streams to ensure the safety of these materials before use as feed substrate. We also advise vigilance in checking incoming feed materials for insecticides with the same MoAs–until further research can rule out negative effects of these other insecticides at legally allowed levels. The results of this study indicate that the potential effect of insecticide residues on farmed insects is a factor that should be taken into consideration when insecticides are approved or MRLs are set.

## Conclusion

Three of the six (chlorpyrifos, propoxur, and tebufenozide) tested insecticides tested at concentrations equal to the respective MRL and 10x that concentration did not affect survival or biomass growth of BSFL. Cypermethrin and spinosad reduced survival and increase of biomass of the BSFL in this experiment. The synergist piperonyl butoxide (PBO) alone did not affect BSFL at concentrations up to 6.0 mg/kg–but when combined with cypermethrin, the negative effects of cypermethrin on growth and survival tended to be enhanced. To validate this, the experiment should be repeated with a higher number of replicates. Imidacloprid stimulated the growth of BSFL, but the exact underlying mechanism for this is unclear and requires more research.

None of the tested substances accumulated in BSFL. This suggests that rearing BSFL on feed containing these insecticides at concentrations equal to the respective MRLs, will not result in exceedance of those limits in BSFL at the point of harvest–notwithstanding the aforementioned risks for survival and growth.

## Supporting information

S1 Table MS/MS conditions. First quadrupole; Q3: Third quadrupole; DP: Declustering potential; CE: Collision energy; CXP: Cell exit potential.
(PDF)

S2 Table Quality control results analytical procedure for Exp. 1: Substrate. a: Relative standard deviation.
(PDF)

S3 Table Quality control results analytical procedure for Exp. 1 and 2: Residual material. *: Solvent only; #: Positive blank; a: Relative standard deviation.
(PDF)

S4 Table Quality control results analytical procedure for Exp. 1 and 2: Larvae. * Solvent only; a: Relative standard deviation.
(PDF)

S5 Table Overview of results for survival (n, number of larvae surviving) and increase in biomass (g) of black soldier fly larvae (Hermetia illucens) for Exp. 1 and Exp. 2. Mean and standard deviation.
(PDF)

S6 Table Analysed concentrations of the compounds in substrate, larvae and residual material (consisting of larval excreta + residual feed) in Exp. 1 (mg/kg). Mean and standard deviation (n = 3). <LOQ: Below level of quantification (LOQ value indicated in brackets). POS: Positive value for the concentration, but could not be quantified (LOQ value indicated in brackets). [1] Mean of two values.
(PDF)

S7 Table Analysed concentrations of compounds in larvae and residual material (consisting of larval excreta + residual feed) and spiked concentrations in substrate in Exp. 2 (mg/kg). Mean and standard deviation (n = 3). [1]: Due to a dilution error in sample preparation, the cypermethrin concentration in the excreta in Exp. 2 could not be quantified. POS: Positive value for the concentration but could not be quantified (LOQ value indicated in brackets).
(PDF)
