# Supplementary material for: Effects of insecticides on mortality, growth and bioaccumulation in black soldier fly (Hermetia illucens) larvae
Source: PLoS One. 2021 Apr 21;16(4):e0249362. doi: 10.1371/journal.pone.0249362 (PMC8059818; doi:10.1371/journal.pone.0249362)
Supplement: S1 Table — First quadrupole; Q3: Third quadrupole; DP: Declustering potential; CE: Collision energy; CXP: Cell exit potential. (PDF) [file pone.0249362.s001.pdf]

S1 Table. MS/MS conditions.

Q1	Q3	Substance ID	DP	CE	CXP
349.9	96.9	Chlorpyrifos	41	41	20
349.9	198	Chlorpyrifos 2	41	25	20
359.9	98.9	Chlorpyrifos D10 1	41	41	20
433.1	191	Cypermethrin	21	21	25
433.1	127	Cypermethrin 2	21	39	25
256.1	175.1	Imidacloprid	41	25	12
256.1	209.1	Imidacloprid 2	41	23	14
260.1	179.1	Imidacloprid D4	41	25	12
260.1	213.1	Imidacloprid D4 2	41	23	14
356.2	177.1	Piperonyl butoxide	31	19	25
356.2	119.1	Piperonyl butoxide 2	31	47	25
210.2	111	Propoxur	61	19	14
210.2	168.1	Propoxur 2	61	13	4
217.2	112	Propoxur D7	61	19	14
732	142	Spinosyn A	186	41	20
732	98	Spinosyn A 2	186	93	22
746	142	Spinosyn D	186	43	20
746	99	Spinosyn D 2	186	75	12
353	133	Tebufenozide	86	33	20
353	297	Tebufenozide 2	86	13	32
362	133	Tebufenozide D9	86	33	20

Legend: Q1: first quadrupole; Q3: third quadrupole; DP: declustering potential; CE: collision energy; CXP: cell exit potential
