# Supplementary material for: Effects of insecticides on mortality, growth and bioaccumulation in black soldier fly (Hermetia illucens) larvae
Source: PLoS One. 2021 Apr 21;16(4):e0249362. doi: 10.1371/journal.pone.0249362 (PMC8059818; doi:10.1371/journal.pone.0249362)
Supplement: S2 Table — a: Relative standard deviation. (PDF) [file pone.0249362.s002.pdf]

S2 Table. Quality control results analytical procedure for Exp. 1: substrate.

Exp. 1 (1*MRL)				
**Substance name**	**Average recovery**	**RSD [a]**	**n**	**Spike range (mg/kg)**
Chlorpyrifos	101%	19%	3	0.005-0.10
Cypermethrin	91%	0.4%	2	0.05-0.10
Imidacloprid	86%	26%	3	0.005-0.10
PBO	115%	6.8%	2	0.005-0.10
Propoxur	138%	2.3%	3	0.005-0.10
Spinosad	85%	7.6%	3	0.005-0.10
Tebufenozide	89%	7.8%	3	0.005-0.10

Legend: a: relative standard deviation.
