# Supplementary material for: Effects of insecticides on mortality, growth and bioaccumulation in black soldier fly (Hermetia illucens) larvae
Source: PLoS One. 2021 Apr 21;16(4):e0249362. doi: 10.1371/journal.pone.0249362 (PMC8059818; doi:10.1371/journal.pone.0249362)
Supplement: S3 Table — *: Solvent only; #: Positive blank; a: Relative standard deviation. (PDF) [file pone.0249362.s003.pdf]

S3 Table. Quality control results analytical procedure for Exp. 1 and 2: residual material.

Exp. 1 (1*MRL)				
**Substance name**	**Average recovery**	**RSD [a]**	**n=**	**Spike range (mg/kg)**
Chlorpyrifos	103%	4.6%	8	0.005
Cypermethrin	#	#	#	0.005
Imidacloprid	97%	11%	8	0.005
PBO	#	#	#	0.005
Propoxur	98%	4.4%	8	0.005
Spinosad	92%	23%	5	0.005
Tebufenozide	111%	15%	8	0.005
**Exp. 2 (+/-*MRL)**				
Chlorpyrifos	84%	7.6%	4	0.005-0.025
Cypermethrin	79%*	31%	2	0.005-0.025
Imidacloprid	101%	9.9%	4	0.005-0.025
PBO	76%	40%	2	0.005-0.025
Propoxur	103%	6.1%	4	0.005-0.025
Spinosad	47%	9.8%	3	0.005-0.025
Tebufenozide	79%	23%	4	0.005-0.025

Legend: *: Solvent only; #: Positive blank; a: Relative standard deviation.
