# Supplementary material for: Effects of insecticides on mortality, growth and bioaccumulation in black soldier fly (Hermetia illucens) larvae
Source: PLoS One. 2021 Apr 21;16(4):e0249362. doi: 10.1371/journal.pone.0249362 (PMC8059818; doi:10.1371/journal.pone.0249362)
Supplement: S4 Table — * Solvent only; a: Relative standard deviation. (PDF) [file pone.0249362.s004.pdf]

S4 Table. Quality control results analytical procedure for Exp. 1 and 2: larvae.

Exp. 1 (1*MRL)				
**Substance name**	**Average recovery**	**RSD [a]**	**n=**	**Spike range (mg/kg)**
Chlorpyrifos	83%	8.1%	4	0.005
Cypermethrin	116%	10%	6	0.005
Imidacloprid	117%	3.8%	6	0.005
PBO	64%	8.2%	6	0.001
Propoxur	94%	1.7%	6	0.001
Spinosad	78%	15%	4	0.001
Tebufenozide	71%	33%	6	0.001
**Exp. 2 (+/-*MRL)**				
Chlorpyrifos	77%	11%	4	0.005-0.025
Cypermethrin	105%*)	6.6%	2	0.005-0.025
Imidacloprid	98%	3.6%	4	0.005-0.025
PBO	94%	2.6%	2	0.005-0.025
Propoxur	91%	3.5%	4	0.005-0.025
Spinosad	98%	14%	3	0.005-0.025
Tebufenozide	120%	5.5%	4	0.005-0.025

Legend: * Solvent only; a: Relative standard deviation
