# Supplementary material for: Effects of insecticides on mortality, growth and bioaccumulation in black soldier fly (Hermetia illucens) larvae
Source: PLoS One. 2021 Apr 21;16(4):e0249362. doi: 10.1371/journal.pone.0249362 (PMC8059818; doi:10.1371/journal.pone.0249362)
Supplement: S5 Table — Mean and standard deviation. (PDF) [file pone.0249362.s005.pdf]

**S5 Table. Overview of results for survival (n, number of larvae surviving) and increase in biomass (g) of black soldier fly larvae (*Hermetia illucens*) for Exp. 1 and Exp. 2. Mean and standard deviation.**

| Substance name(s) | Survival (n) | | Increase in biomass (g) | |
|---|---|---|---|---|
| | Exp. 1 (1*MRL) | Exp. 2 (+/-*MRL) | Exp. 1 (1*MRL) | Exp. 2 (+/-*MRL) |
| Control (blank) | 100.0 ± 0 | - | 11.1 ± 0.3 | - |
| Control + MeOH | 98.7 ± 0.6 | 99.0 ± 1.7 | 10.8 ± 0.3 | 11.0 ± 0.3 |
| Control + ACN | 99.7 ± 0.6 | 99.0 ± 1.0 | 10.8 ± 0.4 | 11.3 ± 0.5 |
| Chlorpyrifos | 99.3 ± 1.2 | 97.0 ± 2.6 | 10.9 ± 0.2 | 11.1 ± 1.0 |
| Propoxur | 97.3 ± 3.1 | 99.3 ± 0.6 | 10.6 ± 0.3 | 11.0 ± 0.1 |
| Imidacloprid | 99.3 ± 0.6 | 100.0 ± 0.0 | 13.0 ± 0.2 | 12.3 ± 0.6 |
| Spinosad | 38.3 ± 10.8 | 98.3 ± 2.9 | 3.4 ± 1.0 | 11.2 ± 0.3 |
| Tebufenozide | 99.3 ± 1.2 | 99.3 ± 0.6 | 10.9 ± 0.1 | 11.2 ± 0.6 |
| Cypermethrin | 87.3 ± 4.0 | 97.7 ± 3.2 | 8.2 ± 0.4 | 10.5 ± 0.1 |
| Piperonyl butoxide | 98.7 ± 1.2 | 99.7 ± 0.6 | 10.9 ± 0.0 | 11.8 ± 0.4 |
| Cyperm. + PBO | 77.7 ± 2.5 | 100.0 ± 0.0 | 4.5 ± 0.5 | 11.2 ± 0.7 |
