# Supplementary material for: Effects of insecticides on mortality, growth and bioaccumulation in black soldier fly (Hermetia illucens) larvae
Source: PLoS One. 2021 Apr 21;16(4):e0249362. doi: 10.1371/journal.pone.0249362 (PMC8059818; doi:10.1371/journal.pone.0249362)
Supplement: S6 Table — Mean and standard deviation (n = 3). <LOQ: Below level of quantification (LOQ value indicated in brackets). POS: Positive value for the concentration, but could not be quantified (LOQ value indicated in brackets). [1] Mean of two values. (PDF) [file pone.0249362.s006.pdf]

**S6 Table. Analysed concentrations of the compounds in substrate, larvae and residual material (consisting of larval excreta + residual feed) in Exp. 1 (mg/kg). Mean and standard deviation (n = 3).**

| **Substance name(s)** | **Analysed concentration in substrate (mg/kg)** | **Analysed concentration larvae (mg/kg)** | **Analysed concentration residual material (mg/kg)** |
|---|---|---|---|
| Chlorpyrifos | 0.03 | POS (<0.005) | 0.034 ± 0.004 |
| Propoxur | 0.05 | <LOQ (0.001) | 0.017 ± 0.007 |
| Imidacloprid | 0.1 | POS (<0.005) | 0.036 ± 0.005 |
| Spinosad | 1.4 | 0.122 [1] | 1.731 ± 0.234 |
| Tebufenozide | 0.05 | POS (<0.001) | 0.020 ± 0.004 |
| Cypermethrin | 0.2 | 0.118 ± 0.018 | 0.927 ± 0.047 |
| Piperonyl butoxide | 6.3 | 0.026 ± 0.004 | 1.764 ± 0.136 |
| Cypermethrin +<br>Piperonyl butoxide | 0.3<br>5.6 | 0.043 ± 0.008<br>0.065 ± 0.024 | 0.758 [1]<br>8.654 ± 0.724 |

*<LOQ: below level of quantification (LOQ value indicated in brackets).*

*POS: positive value for the concentration, but could not be quantified (LOQ value indicated in brackets).*

[1] Mean of two values.
