# Supplementary material for: Effects of insecticides on mortality, growth and bioaccumulation in black soldier fly (Hermetia illucens) larvae
Source: PLoS One. 2021 Apr 21;16(4):e0249362. doi: 10.1371/journal.pone.0249362 (PMC8059818; doi:10.1371/journal.pone.0249362)
Supplement: S7 Table — Mean and standard deviation (n = 3). [1]: Due to a dilution error in sample preparation, the cypermethrin concentration in the excreta in Exp. 2 could not be quantified. POS: Positive value for the concentration but could not be quantified (LOQ value indicated in brackets). (PDF) [file pone.0249362.s007.pdf]

S7 Table. Analysed concentrations of compounds in larvae and residual material (consisting of larval excreta + residual feed) and spiked concentrations in substrate in Exp. 2 (mg/kg). Mean and standard deviation (n = 3).

Substance name(s)	Spiked concentration in feed (mg/kg)	Analysed concentration larvae (mg/kg)	Analysed concentration residual material (mg/kg)
Chlorpyrifos	0.5	0.012 ± 0.005	0.153 ±0.068
Propoxur	0.5	POS (<0.001)	0.036 ± 0.006
Imidacloprid	1.0	0.007 ± 0.001	0.446 ± 0.081
Spinosad	0.2	0.005 ± 0.002	0.099 ± 0.027
Tebufenozide	0.5	0.005 ± 0.001	0.066 ±0.010
Cypermethrin	0.1	0.079 ± 0.025	- [1]
Piperonyl butoxide	2.0	0.019 ± 0.007	0.375 ±0.050
Cypermethrin +<br>Piperonyl butoxide	0.1<br>2.0	0.052 ± 0.019<br>0.021 ± 0.001	0.095 ± 0.020<br>0.428 ± 0.049

[1]: Due to a dilution error in sample preparation, the cypermethrin concentration in the excreta in Exp. 2 could not be quantified.

POS: positive value for the concentration but could not be quantified (LOQ value indicated in brackets).
